# Screen Exposure and Childhood Adiposity in Socially Vulnerable Settings: Cross-Sectional Study

**DOI:** 10.2196/77431

**Published:** 2026-07-08

**Authors:** Ana Duarte, Juliana Martins, Maria José Silva, Cláudia Augusto, Rafaela Rosário

**Affiliations:** 1 School of Nursing University of Minho Braga Portugal

**Keywords:** screen-based media use, screen time, children, adiposity, obesity

## Abstract

**Background:**

Screen-based media use among children has been increasing, particularly in lower socioeconomic groups. As this behavior is linked to obesogenic habits, it is crucial to examine the associations between screen-based media use and adiposity in primary schoolchildren, particularly those from socially vulnerable contexts, such as children from the Educational Territories of Priority Intervention program.

**Objective:**

This study aimed to examine the associations between screen-based media use and adiposity in primary schoolchildren from socially vulnerable contexts.

**Methods:**

This study, part of the BeE-school Project, included 735 children (mean age 7.7, SD 1.2 years; n=380, 51.7% boys and n=355, 48.3% girls) from 10 primary schools located in vulnerable contexts in northern Portugal. Researchers recorded weight, height, and waist circumference and calculated BMI *z* scores and the waist-to-height ratio (WHtR). Screen-based media use was reported by parents using the ScreenQ tool, which includes 4 domains (screen access, frequency of use, media content, and caregiver-child coviewing). Sociodemographic and anthropometric data of parents were obtained via a questionnaire. Generalized linear models were applied.

**Results:**

A higher screen-based media use score was associated with higher BMI *z* scores and WHtR (b=0.064, 95% CI 0.034-0.094 and b=0.002, 95% CI 0.001-0.003, respectively) even after adjusting for children’s sex and age and parents’ education and BMI. Significant associations (*P*<.05) were also observed for the domains of screen access, frequency of use, and media content.

**Conclusions:**

Screen-based media use is linked to higher BMI and WHtR in vulnerable children. Reducing screen access, limiting use frequency, and curating media content could improve health outcomes. Interventions for obesity prevention should consider these factors.

**Trial Registration:**

ClinicalTrials.gov NCT05395364; https://clinicaltrials.gov/study/NCT05395364

## Introduction

Children are now growing up in a technological society, with the use of electronic devices being an essential part of their lives [1]. This has led to an increase in personal screen media device use among children and adolescents, providing numerous entertainment options, such as watching videos, gaming, using social media, and communicating with family and friends [2]. This use encompasses both active and passive engagement with various devices and content [1,3].

From a very early age, screen-based media use is a reality, with children spending many more hours per day in screen time than the international recommendation of no more than 2 hours per day for children and adolescents aged 5 to 17 years [4,5]. According to the American Academy of Pediatrics (AAP), for children aged 6 years and older, healthy habits should be encouraged, and screen-based activities should be limited [6]. It is also recommended to turn off all screens during family meals and outings and to turn off and remove screens from bedrooms 30 to 60 minutes before bedtime [6].

In Portugal, the percentage of children aged 6 to 9 years who spend more than 2 hours per day engaging in screen time on weekdays is around 35%, increasing significantly on weekends to approximately 78% [7]. Also, this prevalence tends to decrease in families with higher levels of parental education, in contrast to an increase in families with lower levels of parental education. In the latter group, children spend around 45% of weekdays and 77% of weekend days exceeding 2 hours of screen time per day [7].

On the other hand, the prevalence of overweight and obesity among school-aged children is 25.7% and 12.5%, respectively, with higher rates observed in families with lower levels of education [7]. This highlights the need to understand the associations between screen-based media use and adiposity in socioeconomically vulnerable contexts.

The associations between excessive screen time and behaviors that promote obesity are well-established [1,8]. For example, obesogenic behaviors (including physical inactivity, increased sedentary behavior, poor dietary habits, and disrupted sleep patterns) [5] have been associated with excessive screen time and childhood obesity [4,5,9]. Additionally, excessive screen time can lead to depression, anxiety, and poor school performance in children [4]. Despite this, there remains controversy over studies’ findings that balance the benefits and harms of screen-based media use, taking into account the type of media and how it is used [4,10]. In addition, although the association between screen use and adiposity has already been studied [8], this relationship remains unclear in the context of primary schoolchildren from socially vulnerable backgrounds.

In Portugal, a school is considered vulnerable if it belongs to the Educational Territories of Priority Intervention. These schools are located in areas characterized by socioeconomic vulnerability, where poverty and social exclusion are prevalent [11,12].

Previous studies examining the relationship between screen-based media use and adiposity in children have shown mixed results. While some report positive associations [1,5,8], others find weaker or inconsistent links depending on factors such as the type of media used and children’s daily contexts [4]. Furthermore, most research focuses on general population samples, leaving limited evidence on how these associations operate among socially vulnerable groups. As a result, important gaps remain in understanding how screen use relates to adiposity in children living in disadvantaged settings.

To address this, the objective of this study was to analyze the associations between screen-based media use and adiposity in primary schoolchildren from socially vulnerable contexts.

## Methods

### Study Design and Participants

This study is part of the broader BeE-school Project, a trial conducted in primary schools that are part of the Educational Territories of Priority Intervention project, which covers territories characterized by social vulnerabilities. For this cross-sectional study, a total of 735 children from 10 primary schools participated with parental consent. The inclusion criteria required that children be from 10 primary schools located in economically and socially disadvantaged areas marked by poverty and social exclusion. Children with cognitive or physical impairments that could compromise data collection were excluded from the study.

### Anthropometrics

Children's weight and height were measured at school by trained researchers using standardized procedures. Measurements were taken with a pediatric scale and stadiometer (SECA 799; SECA GmbH & Co KG), with weight rounded to the nearest 100 g. Height was measured with the head positioned in the Frankfort plane and recorded to the nearest 0.1 cm. Waist circumference was measured directly on the skin, 1 cm above the uppermost lateral border of the right ilium, at the end of normal expiration, and recorded to the nearest 0.1 cm [13]. Children were measured while at school, lightly dressed and without shoes. If there was any doubt about the measurement, the procedure was repeated.

The BMI was calculated as the ratio of body weight to height squared (kg/m^2^) and converted into SD scores (BMI *z* score [BMIz]) adjusted for specific age and sex categories using the World Health Organization (WHO) growth reference [14]. The waist-to-height ratio (WHtR) was calculated by dividing waist circumference by height.

Weight status was defined according to WHO cutoff points of BMI and categorized into 3 groups: normal and underweight (SD ≤1), overweight (1< SD ≤2), and obesity (SD >2). Normal and underweight were grouped together due to the low number of underweight cases in our sample.

Parents self-reported their height and weight, from which BMI was calculated. Parental BMI was categorized according to WHO guidelines into 3 categories: normal and underweight (BMI ≤24.9 kg/m^2^), overweight (BMI 25-29.9 kg/m^2^), and obesity (BMI ≥30 kg/m^2^). Due to the small number of underweight parents (8 mothers and 2 fathers), they were grouped with normal weight cases.

BMI and WHtR were used as indirect anthropometric indicators commonly used to estimate general and central adiposity, acknowledging that they do not represent direct measures of body fat.

### Sociodemographic Information

Parents’ education levels were collected through a questionnaire and categorized into 3 groups: “less than secondary level,” “secondary educational level,” and “university studies.” These variables (mothers’ and fathers’ education levels) were considered as covariates in the fully adjusted model of the analysis.

### Screen-Based Media Use

Children’s screen-based media use was assessed through the ScreenQ questionnaire, filled out by parents. This 16-item questionnaire included 4 domains derived from aspects of media use cited in AAP recommendations: access to screens, frequency of use, media content, and caregiver-child coviewing [15,16].

The evaluation of screen media access includes questions about screen use during meals, in the bedroom, while waiting, ownership of media devices, and screen use at night during the school period (questions 1 to 5). Frequency of use is assessed not only by the number of screen-time hours per day but also by factors such as the age at which children first had contact with screens, whether they use screens to fall asleep, and whether they frequently use screens to calm down (questions 6 to 9). Media content is evaluated through items that assess exposure to violent content, content pacing, and whether children independently choose what to view (questions 10 to 12). Finally, caregiver-child coviewing considers not only the presence of an adult but also the nature of interaction between the adult and the child during screen use (questions 13 to 15) [16].

The 16 items of ScreenQ are summed for a total score ranging from 0 (perfect adherence with AAP recommendations) to 27 points (extreme nonadherence—a major risk for adverse effects). The score for each dimension was calculated by summing the scores of the corresponding questions.

Internal consistency of the scale for children aged >5 years is 0.64 (Cronbach α), which indicates a good internal consistency [15]. In our sample, Cronbach α was also calculated, and a value of 0.61 was obtained, indicating good internal consistency. The total score and the scores for each domain were analyzed as predictors of adiposity in the statistical analysis.

### Statistical Analysis

Descriptive analysis encompassed measures of central tendency and dispersion tailored to each variable type. Quantitative variables were summarized using mean and SD, while categorical variables were described using counts (n) and percentages (%). Proportions of categorical variables were compared using chi-square tests for contingency tables, and a 2-tailed Student *t* test was applied for quantitative variables.

Generalized linear models were used to assess the associations between children’s screen-based media use (predictor) and children’s adiposity (BMI and WHtR) as dependent variables, with a significance level set at .05.

Three models were used. Model 1 was the unadjusted model, model 2 incorporated children’s sex and age as covariates, and model 3 represented the fully adjusted model, including children’s sex and age, as well as mothers’ and fathers’ education levels, and mothers’ and fathers’ BMI as covariates. Data analysis was conducted using SPSS (version 29.0.1; IBM Corp).

### Ethical Considerations

This study was approved by the Ethics Committee for Life and Health Sciences (CEICVS 009/2022) of the University of Minho and registered on the ClinicalTrials database (NCT05395364). The research adhered to the Code of Ethics of the World Medical Association (Declaration of Helsinki). All participants, including parents or caregivers, signed informed consent forms, and children were asked for their assent to participate before any procedures were conducted.

## Results

Descriptive data for the participants are presented in Table 1. In a sample of 735 school-aged children, 380 (51.7%) were boys and 355 (48.3%) were girls, with a mean age of 7.7 (SD 1.2) years. Most of the children had normal weight (448/728, 61.5%), 22.7% (165/728) were overweight, and 15.8% (115/728) had obesity. Around half of the mothers (305/552, 55.3%) had normal weight, whereas 37.8% (191/505) of fathers fell into this category. Nearly half of fathers were overweight (237/505, 46.9%).

**Table 1:** Descriptive analysis^a^.

		All (n=735)	Boys (n=380)	Girls (n=355)	*P* value	
Age (years), mean (SD)		7.7 (1.2)	7.7 (1.2)	7.7 (1.2)	.67	
BMI *z* score		0.69 (1.30)	0.76 (1.36)	0.62 (1.23)	.15	
**BMI category, n (%)**						.16
	Normal and underweight	448 (61.5)	234 (62.2)	214 (60.8)		
	Overweight	165 (22.7)	76 (20.2)	89 (25.3)		
	Obesity	115 (15.8)	66 (17.6)	49 (13.9)		
Waist-to-height ratio, mean (SD)		0.47 (0.05)	0.47 (0.05)	0.47 (0.05)	.58	
ScreenQ total score, mean (SD)		10.27 (4.02)	10.64 (3.94)	9.88 (4.09)	.04	
**Mothers’ education, n (%)**						.25
	Less than secondary level	105 (17.3)	46 (15)	59 (19.6)		
	Secondary educational level	234 (38.6)	117 (38.2)	117 (38.9)		
	University studies	268 (44.2)	143 (46.7)	125 (41.5)		
**Fathers’ education, n (%)**						.03
	Less than secondary level	168 (29.1)	75 (26.2)	93 (32)		
	Secondary educational level	223 (38.6)	104 (36.4)	119 (40.9)		
	University studies	186 (32.2)	107 (37.4)	79 (27.1)		
**Mothers’ BMI category, n (%)**						.75
	Normal and underweight	305 (55.3)	155 (56.6)	150 (54)		
	Overweight	169 (30.6)	83 (30.3)	86 (30.9)		
	Obesity	78 (14.1)	36 (13.1)	42 (15.1)		
**Fathers’ BMI category, n (%)**						.62
	Normal and underweight	191 (37.8)	95 (37.8)	96 (37.8)		
	Overweight	237 (46.9)	114 (45.4)	123 (48.4)		
	Obesity	77 (15.2)	42 (16.7)	35 (13.8)		

^a^Percentages were calculated using the available data for each variable; therefore, denominators may vary across categories due to missing data.

Tables 2 and 3 present the associations between children’s screen-based media use and adiposity (with BMIz as the surrogate measure of adiposity in Table 2 and WHtR in Table 3). We conducted the analysis using 3 models to explore these associations.

**Table 2:** Association between children’s screen-based media use and their BMI z score^a^.

		Model 1^b^, B (95% CI)	Model 2^c^, B (95% CI)	Model 3^d^, B (95% CI)
**ScreenQ total score**		0.064 (0.034 to 0.094)^e^	0.059 (0.029 to 0.089)^e^	0.053 (0.023 to 0.083)^e^
	Access	0.105 (0.048 to 0.163)^e^	0.097 (0.040 to 0.155)^e^	0.064 (0.005 to 0.122)^e^
	Frequency	0.098 (0.027 to 0.169)^e^	0.095 (0.024 to 0.166)^e^	0.078 (0.004 to 0.151)^e^
	Content	0.140 (0.059 to 0.221)^e^	0.117 (0.032 to 0.203)^e^	0.119 (0.034 to 0.204)^e^
	Coviewing	0.002 (–0.081 to 0.085)	–0.007 (–0.090 to 0.076)	0.008 (–0.075 to 0.092)

^a^Values are presented as unstandardized regression coefficients (B) with corresponding 95% CI.

^b^Model 1: unadjusted model.

^c^Model 2: adjusted model for children’s sex and age.

^d^Model 3: adjusted model for children’s sex and age, mothers’ and fathers’ education level, and mothers’ and fathers’ BMI.

^e^*P* value<.05.

**Table 3:** Association between children’s screen-based media use and waist-to-height ratioa.

		Model 1^b^, b (95% CI)	Model 2^c^, b (95% CI)	Model 3^d^, b (95% CI)
**ScreenQ total score**		0.002 (0.001 to 0.003)^e^	0.002 (0.001 to 0.003)^e^	0.002 (0.001 to 0.003)^e^
	Access	0.004 (0.002 to 0.006)^e^	0.004 (0.002 to 0.006)^e^	0.004 (0.002 to 0.006)^e^
	Frequency	0.004 (0.001 to 0.006)^e^	0.004 (0.001 to 0.006)^e^	0.004 (0.001 to 0.006)^e^
	Content	0.005 (0.002 to 0.009)^e^	0.005 (0.002 to 0.009)^e^	0.005 (0.002 to 0.009)^e^
	Coviewing	–0.0004 (–0.004 to 0.003)	–0.0004 (–0.004 to 0.003)	–0.0004 (–0.004 to 0.003)

^a^Values are presented as unstandardized regression coefficients (B) with corresponding 95% CI.

^b^Model 1: unadjusted model.

^c^Model 2: adjusted model for children’s sex and age.

^d^Model 3: adjusted model for children’s sex and age, mothers’ and fathers’ education level, and mothers’ and fathers’ BMI.

^e^*P* value<.05.

A higher screen-based media use score was significantly associated with higher BMIz (b=0.053, 95% CI 0.023-0.083) and WHtR (b=0.002, 95% CI 0.001-0.003) in the fully adjusted model. Similar associations were observed for the domains of screen access, frequency of use, and media content. Caregiver-child coviewing was not significantly associated with BMIz (b=0.008, 95% CI –0.075 to 0.092) or WHtR (b=–0.0004, 95% CI –0.004 to 0.003).

## Discussion

### Principal Results

We found that children with higher ScreenQ scores—reflecting screen media use that does not align with AAP recommendations—tended to have higher BMIz and WHtR values. We also found that specific characteristics of screen media use—such as screen access, frequency of use, and media content—were significantly associated with both measures.

### Comparison With Prior Work

Our findings indicate that children with nonadherence to AAP recommendations had a higher risk of adverse effects concerning surrogates of obesity. This is in accordance with existing evidence that identifies overweight and obesity as one of the first health consequences of excessive screen time in children [1,4,17,18]. Since 1985, when the first paper about the effects of media use on children’s adiposity was published by Dietz and Gortmaker [19], the scientific community has observed the negative consequences of excessive media use by children. Additionally, the frequency of media use has been increasing in recent years, particularly during successive lockdowns when screen use significantly surged in many regions worldwide [20]. The long-term effects of the COVID-19 pandemic lockdowns remain unclear [21], and it is possible they have influenced the frequency of postpandemic screen viewing.

According to Hutton et al [16], screen media use could pose potential neurobiological risks. Furthermore, the AAP has cited language delay, poor sleep, impaired executive function and general cognition, and decreased parent-child engagement as some of the other consequences of screen-based media use by children [16,22].

Screen time is typically associated with sedentary behavior and linked to a higher consumption of energy-dense foods, promoting an energy imbalance [23-25]. Children who increased or consistently maintained high screen time from preschool to school age were more likely to adopt unhealthier dietary patterns, such as frequent snacking or consuming energy-dense foods [26]. Media can act as a trigger or prompt for eating, extend the duration of eating, or distract from feelings of satiety [8]. Another growing concern is the presence of food marketing in digital media, especially targeted at children, which can explain the link between screen-based media use and higher energy consumption [8]. Marketing and the commercial determinants of health have the power to shape children's food preferences, purchasing behaviors, and consumption patterns. Because the most commonly promoted food products to children are sugary breakfast cereals, soft drinks, savory snacks, confectionery, and fast food, accounting for about 60% to 90% of all food marketing [27], it is possible that their consumption might increase. Screen-based media use is also linked to poorer sleep, which can cause changes in appetite-regulating hormones such as ghrelin and leptin, leading to increased hunger and decreased satiety [8].

Concerning screen access, children now have a wide variety of screen media devices available to them, such as smartphones, tablets, laptops, desktops, gaming consoles, and televisions. According to Dy et al [28], the devices that children have the most access to are televisions, followed by smartphones and tablets. With all these access opportunities, screen time during important periods such as mealtimes and bedtimes has emerged, with screen use during meals becoming increasingly common among children and adolescents [29]. This has a significant impact on child health, as screen use during meals has been linked to increased consumption of snacks, including high-calorie and low-nutrient products [29].

The type of content is another important aspect to discuss. Screen media content available to children varies widely, differing in aspects such as target audience, educational value, and language used [30]. These factors have been linked to cognitive and learning outcomes [28,30,31]. Content can also influence diet, particularly through the promotion of unhealthy eating behaviors via junk food marketing [17]. Children are exposed to marketing of unhealthy foods and are considered a vulnerable group for this type of digital content [17,30]. However, depending on the content, media use can also have a significantly positive impact on children’s health, and interventions that use media to promote the adoption of healthy lifestyles should be considered [1,31].

Despite some studies identifying coviewing screen media as having a positive impact on health outcomes [28], in our study, this dimension did not appear to be significantly associated with adiposity. According to Dy et al [28], children are nearly 9 times more likely to have excessive screen time when watching alone compared to watching with an adult, and excessive screen use has been linked to worse health outcomes, as mentioned earlier. However, our findings regarding this specific domain could be related to the type of coviewing, such as interactive or passive coviewing. Alroqi et al [30], who investigated the influence of the quantity, content, and context of screen media use on the language development of 85 Saudi children aged 1 to 3 years, reported that passive coviewing of television was more common than interactive coviewing, with the latter appearing to have a positive effect on children’s development and health. It is noteworthy that despite the nonsignificant result, the association between coviewing and BMIz is positive, and a higher screen use without adult supervision is associated with a higher BMIz.

Apart from health consequences, media use by children was also identified by Priftis and Panagiotakos [4], in a review documenting the health effects of excessive screen viewing on children and adolescents, as potentially having a socioeconomic impact as well. Moreover, lower maternal education levels and lower household income were linked to higher odds of increased screen time [26]. Moreover, some parents seem to believe that screen time increases children’s creativity and imagination [28], while others reported having strict rules concerning screen-based media use in their homes [10].

This study was conducted with a specific group of children from primary schools located in socially vulnerable territories. However, not all families had a very low socioeconomic status, which may explain the relatively higher rates of mothers (268/607, 44.2%) and fathers (186/577, 32.2%) with a university education level. Nevertheless, as educational level is considered an important surrogate variable for socioeconomic status [32], we accounted for it as a potential confounder in our analysis.

This study has several strengths that should be emphasized. First, we used trained researchers to collect the anthropometric measures, and we included both BMIz and WHtR as measures of adiposity, thereby enhancing the validity of our findings. To assess adiposity, BMI has been widely used as a surrogate measure; however, WHtR has recently been identified as a potentially better surrogate measure for adiposity. It predicts total and trunk fat mass more accurately and can discriminate lean mass in the pediatric population, unaffected by children’s age and sex [33]. Second, we used the ScreenQ as a measure of screen-based media use, an instrument that has already been validated for Portuguese children. ScreenQ evaluates not only the screen time but also other dimensions of screen-based media use: access to screens, frequency of use, media content, and caregiver-child coviewing [15,16], allowing a more comprehensive analysis. Third, the analysis considered potential confounders such as children’s characteristics and parents’ sociodemographic and weight status. Finally, this study was conducted in a specific context of vulnerability, which has not yet been extensively studied in Portugal.

### Limitations

We recognize that this study has some limitations. First, the cross-sectional design did not allow us to identify a causal relationship. Second, the data on ScreenQ, sociodemographic factors, and parents’ anthropometrics were self-reported by parents, potentially introducing social desirability bias. To minimize the impact of these limitations on the credibility of the findings, we used validated instruments (such as the ScreenQ) and ensured anonymity in participation to reduce potential response bias. We also interpreted the results cautiously, clearly acknowledging the direction of the associations without implying causality. These measures help safeguard the trustworthiness and robustness of the study’s conclusions.

Finally, the use of BMI and WHtR as surrogate indicators of adiposity represents a limitation, as these measures may not fully capture body fat distribution and can be influenced by factors such as muscle mass. Although BMI and WHtR likely capture relevant population-level variation in body shape [34], future studies should consider incorporating objective measures of adiposity to provide more precise assessments.

### Conclusions

Screen-based media use was significantly associated with higher BMI and WHtR among primary schoolchildren from socially vulnerable contexts. Future school-based interventions should emphasize the substantial impact of screen use on children’s adiposity, particularly in light of their socioeconomic circumstances.

## Data Availability

The datasets generated and/or analyzed during this study are available from the corresponding author on reasonable request.

## Abbreviations

AAP: American Academy of Pediatrics
BMIz: BMI z score
WHO: World Health Organization
WHtR: waist-to-height ratio
